# Banding cytogenetics of chimeric hybrids *Coturnixcoturnix* × *Coturnixjaponica* and comparative analysis with the domestic fowl

**DOI:** 10.3897/CompCytogen.v12i4.27341

**Published:** 2018-10-16

**Authors:** Yasmine Kartout-Benmessaoud, Kafia Ladjali-Mohammedi

**Affiliations:** 1 University of Sciences and Technology Houari Boumediene, Faculty of Biological Sciences, Laboratory of Cellular and Molecular Biology, Team of Developmental Genetics. USTHB, PO box 32 El-Alia, Bab-Ezzouar, 16110 Algiers, Algeria University of Sciences and Technology Houari Boumediene Bab-Ezzouar Algeria; 2 University of Bejaia, Faculty of Nature and Life Sciences, Department of Physico-Chemical Biology, 06000, Bejaia, Algeria University of Bejaia Bejaia Algeria

**Keywords:** Avian cytogenetics, cell culture, chimeric hybrids, *Coturnixcoturnix* × *Coturnixjaponica*, GTG-banding, intrachromosomal rearrangements.

## Abstract

The Common quail *Coturnixcoturnix* Linnaeus, 1758 is a wild migratory bird which is distributed in Eurasia and North Africa, everywhere with an accelerating decline in population size. This species is protected by the Bonn and Berne conventions (1979) and by annex II/1 of the Birds Directive (2009). In Algeria, its breeding took place at the hunting centre in the west of the country. Breeding errors caused uncontrolled crosses between the Common quail and Japanese quail *Coturnixjaponica* Temminck & Schlegel, 1849. In order to help to preserve the natural genetic heritage of the Common quail and to lift the ambiguity among the populations of quail raised in Algeria, it seemed essential to begin to describe the chromosomes of this species in the country since no cytogenetic study has been reported to date. Fibroblast cultures from embryo and adult animal were initiated. Double synchronization with excess thymidine allowed us to obtain high resolution chromosomes blocked at prometaphase stage. The karyotype and the idiogram in GTG morphological banding (G-bands obtained with trypsin and Giemsa) corresponding to larger chromosomes 1–12 and ZW pair were thus established. The diploid set of chromosomes was estimated as 2N=78. Cytogenetic analysis of expected hybrid animals revealed the presence of a genetic introgression and cellular chimerism. This technique is effective in distinguishing the two quail taxa. Furthermore, the comparative chromosomal analysis of the two quails and domestic chicken *Gallusgallusdomesticus* Linnaeus, 1758 has been conducted. Differences in morphology and/or GTG band motifs were observed on 1, 2, 4, 7, 8 and W chromosomes. Neocentromere occurrence was suggested for Common quail chromosome 1 and Chicken chromosomes 4 and W. Double pericentric inversion was observed on the Common quail chromosome 2 while pericentric inversion hypothesis was proposed for Chicken chromosome 8. A deletion on the short arm of the Common quail chromosome 7 was also found. These results suggest that Common quail would be a chromosomally intermediate species between Chicken and Japanese quail. The appearance of only a few intrachromosomal rearrangements that occurred during evolution suggests that the organization of the genome is highly conserved between these three galliform species.

## Introduction

Birds represent a class of tetrapod vertebrates which contains a vast diversified variety of species (Jarvis et al. 2014). Extensive studies regarding birds are undertaken by researchers in Developmental Biology and Animal Cytogenetics, with over 1000 avian karyotypes published. However, few of them were deeply and accurately analyzed by using the chromosome banding. This results from difficulty of analysis in cell culture and establishment of chromosome issues (Christidis 1990).

The avian genome is characterized by very high chromosome number, with an average of 2N=76 - 80 (Werner 1927, Bed’Hom et al. 2003). The sex determination is of type ZZ for the homogametic male (equivalent to human XX), and ZW for the heterogametic female (equivalent to human XY). Besides the macrochromosomes which are easily identifiable, the microchromosomes are almost indistinguishable one from another (Masabanda et al. 2004, Griffin et al. 2007, Griffin and Burt 2014, Graves 2014). That is why mostly bird karyotypes are analyzed partially and limited to the few macrochromosomes (Shibusawa et al. 2004). Despite their small physical size, microchromosomes encode 50% of genes and are characterized by high CpG islands content and an early replicating pattern (Dutrillaux 1986, McQueen et al. 1996, Rodionov 1996, Burt 2002, Skinner et al. 2009, Hansmann et al. 2009).

Taxonomically, the majority of avian karyotypes are exceptionally stable and present conserved synteny regions (Shetty et al. 1999, Derjusheva et al. 2004, Shibusawa et al. 2004, Nie et al. 2009, Nanda et al. 2011, Ishishita et al. 2014). Birds have experienced fast series of speciation events during millions of years (Nadachowska-Brzyska et al. 2015, Griffin et al. 2015). Although intra-chromosomal rearrangements occur widely, inter-chromosomal ones are rare events estimated as 1.25 per million years (Zhao and Bourque 2009, Zhang et al. 2014, Hooper and Price 2017, Kretschmer et al. 2018). These reshufflings could be the cause or consequence of speciation, or a result of adaptation (Nishida et al. 2008, Völker et al. 2010, Romanov et al. 2014).

Like the domestic fowl *Gallusgallusdomesticus* Linnaeus, 1758, the Common quail *Coturnixcoturnix* Linnaeus, 1758 and the Japanese quail *Coturnixjaponica* Temminck & Schlegel, 1849 are the representative species of the ancestral order Galliformes. The Japanese quail originates from the eastern Palearctic (Siberia, Mongolia, Korea, Northeastern China and Japan) but has lost migratory behavior, normal in its wild type (Del Hoyo et al. 1994). The Japanese quail is reared in Europe as a farm animal for meat and eggs (Minvielle 1998, 2004). On the other hand, the Common quail is a wild migratory bird which is hunted for its scrumptious meat and eggs. It is also called the European quail given its characteristic distribution area. It breeds widely in Central and Southern Europe, as well as in Western Asia and North Africa (Johnsgard 1988). The Common quail shows very important annual fluctuations and it is listed under ‘Least Concern’ in the International Union of Conservation of Nature Red List. Nevertheless, it is protected by several conventions (Bonn and Bern in 1979, appendix II/1 of the birds Directive (2009/147/CE) of the European Parliament) (BirdLife International 2004, Hennache and Ottaviani 2011, Puigcerver et al. 2012).

In Algeria, a global strategy of preservation of the Common quail was organized thanks to collaborations between National research stations and Hunting Centre. The breeding of this species was kept in the form of reduced numbers at the Tlemcen Hunting Centre in the west of the country. The strong phenotypic resemblance between the European and the Japanese quail originated from errors committed during the breeding stage brought about as a result of uncontrolled crossings between these species and the appearance of hybrids (information supplied by the Tlemcen Hunting Centre).

Indeed, the Japanese quail is different from the European quail although they were considered for a long time as two subspecies (Austin and Kuroda 1953, Vaurie 1965, Minvielle 1998). Phylogenetic studies based on the analysis of nucleotide sequences of mitochondrial genes showed that the Japanese quail is of more recent appearance (Nishibori et al. 2001, Huang and Ke 2014).

However, the hybridization has negative consequences on the evolution of the genetic heritage of the species concerned and their preservation (Arnold 1997, Barton 2001). It can be a direct consequence of human activities (Arnold 2004). So, the genetic introgression is a frequent event in closely related animal species (Rhymer and Simberloff 1996, Arnold 1997, Allendorf et al. 2001). Indeed, both taxa of quails are known for their capacity to cross in captivity (Lepori 1964, Dérégnaucourt et al. 2001). During the breeding season, the natural ranges of common and Japanese quail overlap only in the Lake Baikal area (Russia) and in the Kentei region (Mongolia) (Barilani et al. 2005). However, no extensive natural hybridization has been reported (Del Hoyo et al. 1994, Guyomarc’h et al. 1998).

Thus, the introgressive hybridization caused by the uncontrolled release of Japanese quails seems to induce a very worrying genetic shift. In fact, a more or less complete loss of the migratory ability of hybrid subjects has been noted with the appearance of a hybrid song and the assignment of morphological and behavioral characters (Guyomarc’h et al. 1998, Dérégnaucourt and Guyomarc’h 2003, Dérégnaucourt et al. 2005).

Interspecific chimeras can be also met with at an early development stage, resulting from a crossing between closely related species especially in birds (Basrur and Yamashiro 1972). Chimeras are animal bodies stemming from a double fertilization, from an oocyte and from a polar globule, each by a different sperm cell creating two zygotes which would merge in a single embryo. The final result remains the creation of an unprecedented living creature within which different cells, from a genetic point of view, live together (Wolinsky 2007). Indeed, hybrids stemming from related species are often fertile individuals (Asmundson and Lorenz 1957, Makos and Smyth 1970). It is the case of mice *Musmusculus* Linnaeus, 1758 and *Muscaroli* Bonhote, 1902, chicken-quail hybrid and pheasant-turkeys hybrids (Bammi et al. 1966, Benirschke 1967, Rossant et al. 1983). Hybrids stemming from more distant species have reduced fertility or are even sterile as in the crossings mouse - rat and sheep - goat (Polzin et al. 1987, MacLaren et al. 1993).

Although high resolution molecular techniques are well advanced, chromosome banding remains an effective method for delineating chromosome homologies between phylogenetically related species. Indeed, banding colorations allow participation, in an important way, in the studies of taxonomy and phylogenetics and reveal the ancestral chromosome rearrangements of vertebrates (Rumpler and Rumpler 1976, Yunis et al. 1982, Bouayed 2004, Muffato 2010, Ouchia-Benissad and Ladjali-Mohammedi 2018).

The purpose of this study is to establish the karyotype of the Common quail *Coturnixcoturnix* at high resolution level with morphological banding techniques. So far, no study of the chromosomes of this species has been reported. Also, considering the possibility of an introgressive hybridization between the Common quail and the Japanese quail, it was necessary to analyze the individuals expected to be the hybrid animals (*Coturnixcoturnix* × *Coturnixjaponica*) in order to remove the ambiguity within the quail populations bred in Algeria. Comparative chromosome analysis by GTG banding of both species of quails and the domestic fowl *Gallusgallusdomesticus* has been conducted to detect certain rearrangements that would have occurred during speciation and to estimate the degree of conservation between these species.

## Material and methods

### Embryos and Adults

Common quail *Coturnixcoturnix*: Five fertile eggs and an adult, 6-month-old male brought during the reproduction period from the Tlemcen Hunting Centre, Algeria (34°53'24"N, 1°19'12"W) have been analyzed in the present study.

Japanese quail *Coturnixjaponica*: Five fertile eggs resulting from animals raised in the Hunting Centre of Zeralda, Algeria (36°42'06"N, 2°51'47"E) were also cultivated.

Hybrid animals: The Tlemcen Hunting Centre us to analyze eggs resulting from animals expected to be hybrid and resulting of an uncontrolled crossing between the Common quail and the Japanese quail. So, seven fertile eggs obtained at the 15^th^ generation have been cultured.

### Cell cultures

The age of all the embryos put in cultures in the present study varies between 8 and 12 days. The eggs were incubated in a ventilated incubator where the conditions of hygrometry (55%) and temperature (39.5 °C) are maintained. For the embryos and the adult animal, the cellular cultures were carried out under sterile conditions in a chamber of cellular culture equipped with a vertical laminary flow hood (Polaris72 N°19311). The fibroblast primary cultures were carried out after samples were taken from fragments of various body parts (lung, heart, liver, kidneys and muscles). The cells were put in suspension in medium of RPMI 1640 supplemented by 20mm of HEPES, 1% of L-Glutamine (Gibco ref.: 22409-015, batch: 695608), 10% of foetal calf serum (FCS, Gibco ref.: 10270-106, batch: 41Q4074K), Penicillin-Streptomycin 1% and 1% of Fungizone (Gibco ref.: 15160-047, Batch: S25016D). The cells in culture were incubated at 41 °C (Ladjali 1994, Ladjali et al. 1995).

### Chromosomal preparations

Cultures were synchronized with a double thymidine block (10mg /ml, Sigma) during S phase in order to increase the yield of metaphase and early metaphase cells as described by Ladjali et al. (1995). The half-cycle was estimated at 6–7 hours. Cells were treated during 3 to 5 min with colchicine (Sigma: 4μg/ml). Then, cells cultures were harvested by treating them with trypsin (Gibco: 25300-054; 0,05%). After centrifugation, cells were suspended for 13 min at 37 °C in hypotonic solution 1:5 (FCS- Distilled water) supplemented with EDTA or Sodium Citrate. Then, they were fixed in 3:1 (Ethanol:Acetic Acid). Chromosome preparations were dropped onto clean slides, wet with a film of distilled water and air dried. Twelve double synchronizations were performed for each species. We spread about 15 slides per synchronization. Approximately 20 metaphases were analyzed from each individual (embryos, animal).

### Chromosome staining and banding

The method of Seabright (1971) modified by Ladjali et al. (1995) was used to induce GTG bands (G-bands obtained with Trypsin and Giemsa). Chromosome preparations aged from 3 to 10 days were incubated for 12 seconds in a trypsin solution (0.25%) prepared extemporaneously. The preparations were rinsed in phosphates buffer (pH = 7) then colored during 10 to 15min with 6% Giemsa, pH= 6.8 (Batch: BCBF9150; Ref: 48900-1-L-F).

### Microscopy

Chromosome preparations were screened under a photonic microscope (Zeiss Scope A1, Axio) equipped with a digital black-and-white camera (Cool cube 1). Images have been captured by metasystem processing software. Photos were treated by ADOBE PHOTOSHOP 7.0 software.

### Karyotypes

The establishment of karyotypes is based upon nomenclature taking into account the morphology and size of chromosomes according to the International System for Standardized Avian Karyotypes (ISSAK) (Ladjali-Mohammedi et al. 1999).

### Chromosomes measurements

The IMAGE J software was used to integrate the scale bar on the photos (Rueden et al. 2017), and the KARYOTYPE 2.0 software allowed us to measure the relative lengths of chromosomes (Altınordu et al. 2016).

## Results

### Cell Cultures and synchronization

The fibroblast cultures derived from wild quail proved to be very sensitive to the various treatments (thymidine, BrdU and FdU). Indeed, cell culture follow-up showed fibroblast set up after two to five days, but after trypsination (0,05%) and synchronization, most cells died both in the embryos and adult. However, in Japanese quails and hybrids, the cells showed good viability after incorporation of different treatments. The cells from embryos provide a higher mitotic index and a greater potential for cell division compared to adult animal. The mitotic index observed in wild quail averaged one to two metaphases per a field (G×10). On the other hand, in Japanese quails and expected hybrids, the mitotic index was approximately 10 metaphases.

The control of the cell cycle by synchronization seems to be the best and most suitable procedure for blocking the so-called high-resolution chromosomes. The duration of the cell half cycle was estimated at 6 hours for both quail species. The majority of cells, dividing the two quail species, obtained in this experiment were at the metaphase and prometaphase stages.

### Diploid number and GTG-banded karyotype of the Common quail *Coturnixcoturnix*

**Figure 1.**
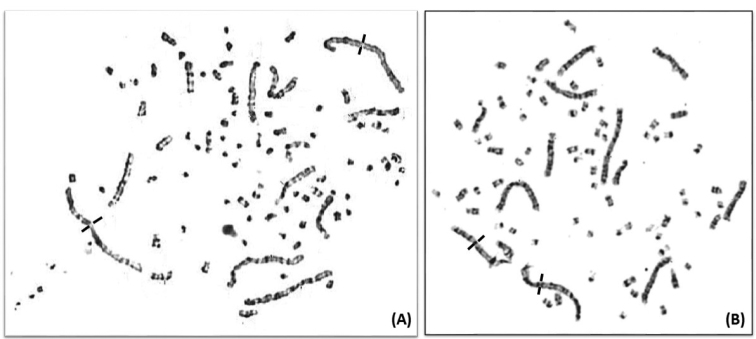
Prometaphase spreads following the GTG-banded chromosomes of **A** the Common quail *Coturnixcoturnix***B** Japanese quail *Coturnixjaponica* (Black bars indicate the centromere positions of the chromosomes 1).

Forty-five metaphases which showed well-distributed chromosomes were selected to count the diploid number of the Common quail, thus estimated at 2N=78 and represented by 38 pairs of autosomes and one pair of sex chromosomes (Figure 1A).

**Figure 2.**
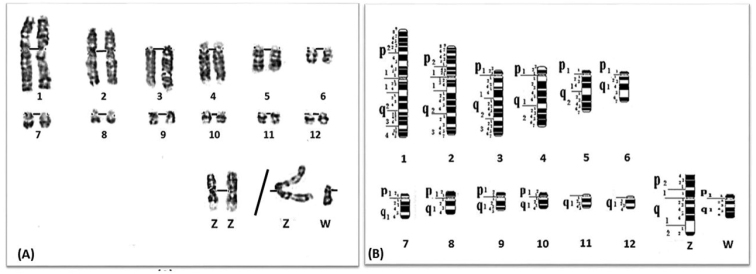
**A** GTG-banded karyotype for pairs 1 to 12 and sex chromosomes of the Common quail *Coturnixcoturnix*. **B** Idiogram corresponding to **A**.

The GTG staining technique revealed clear G-banding patterns in all macrochromosomes and microchromosomes to size number 12 at least. Only the first 12 pairs and ZW sex chromosomes of the Common quail were described in this study (Figure 2A). The ISSAK (1999) will be the basis for chromosome nomenclature. The results of measurements of the relative lengths were also presented (Table 1). The corresponding idiogram was proposed on the basis of the mean of 25 metaphases analyzed. It represents the largest pairs 1–10 (arms p, q) and chromosomes of the lesser size (arm q) of pairs 11–12 (Figure 2B).

**Table 1.** Size of the mitotic chromosomes of the Common quail *Coturnixcoturnix* (n=14) p: short arm, q: long arm, p+q: relative length, CI: Centromeric index=p/(p+q) × 100.

**Chromosomes**	**p (µm)**	**q(µm)**	**q/p**	**p+q(µm)**	**CI%**
**1**	1.71	2.29	1.32	4	42
**2**	1.25	1.66	1.32	2.91	42.95
**3**	0.12	2.15	17.91	2.27	5.28
**4**	0.30	1.85	6.16	2.15	13.95
**5**	0.10	1.40	8.25	1.50	6.7
**6**	0.11	0.9	8.18	1.01	10.89
**7**	0.18	0.79	4.38	0.97	18.55
**8**	0.26	0.51	1.96	0.77	33.76
**9**	0.1	0.65	6.5	0.75	13.33
**10**	0.08	0.58	7.25	0.66	12.12
**11**	0.1	0.55	5.5	0.65	15.38
**12**	0.08	0.48	6	0.56	14.28
**Z**	1.06	1.08	0.49	2.14	49.53
**W**	0.16	0.8	5	0.96	16.66

Chromosomes 1 and 2 are submetacentric. Their arm ratios are quite similar (q/p = 1,32) (Table 1). The p arm of the chromosome 1 has two regions and 15 G-bands. The landmark band 21 divides the p arm into two regions. 19 G-bands are observed on the q arm; three prominent negative bands (21, 31 and 41) divide the arm into four regions. Chromosome 2 has 13 G-bands, one central band (21) which separates the p arm into two regions. Three regions are observed on the q arm with 16 G-bands. Chromosome 3 is acrocentric, it has one region and 3 G-bands on the p arm. The q arm has three regions and 19-G bands. The first region is marked by a prominent negative band (13). Chromosome 4 is subtelocentric. The p arm of the chromosome 4 has one region and 3 G-bands. The q arm has two regions. 11 G-bands; region 2 is marked by a subcentral negative band (21). Chromosomes 5 and 6 are acrocentric. Chromosome 5 has a p arm with one region and 1 G-band; a subcentromeric positive band (11). Two regions are observed on q arm with 11 G-bands. On chromosome 6, one region and one narrow subcentromeric positive G-band (11) are observed on the p arm. The q arm has one region. 7 G-bands, band 17 is a positive in the telomere region, which is not always visible. Chromosome 7 is telocentric. It has one region on the p arm, 2 G-bands. One region on the q arm, 6 G-bands. Chromosome 8 is submetacentric. It has one region and 2 G-bands on the p arm. One region and 4 G-bands are observed on the q arm. On the p arm of the chromosome 9, one region and 2 G-bands, a subcentromeric negative band (11) followed by a positive band (12). The q arm has one region, 5 G-bands. Chromosome 10 has one region observed on the p arm with 2 G-bands. The q arm has one region, 4 G-bands. The q arm of the chromosome 11 has one region and 5 G-bands. Chromosome 12 has one region on the q arm. 4 G-bands are observed, a subcentromeric negative band (11) followed by two prominent positive bands (12 and 14) separated by a large negative (13). Sex chromosomes Z and W are respectively, metacentric and subtelocentric. Chromosome Z has two regions on the p arm. 5 G-bands, region 1 has a large subcentromeric positive band (11). The q arm has two regions. 8G-bands, region 1 has a subcentromeric negative band (11). Band 21 is the characteristic large heterochromatic region. One region and one G-band are observed on the p arm of the chromosome W. The q arm has one region and 6 G-bands.

### Morphometry of the Japanese quail *Coturnixjaponica* chromosomes and GTG-band karyotype

In this study, we confirmed the diploid number of chromosomes of the Japanese quail, 2N=78 (Figure 1B). In general, the karyotype of this species is arranged similarly to that of the previous species. The largest twelve pairs range in size from 4,1µm to 0,53µm (Table 2). These measurements show that chromosomes of Common quail (Table 1) were slightly more decondensed than those of Japanese quail (certainly due to the success of double synchronization).

**Figure 3.**
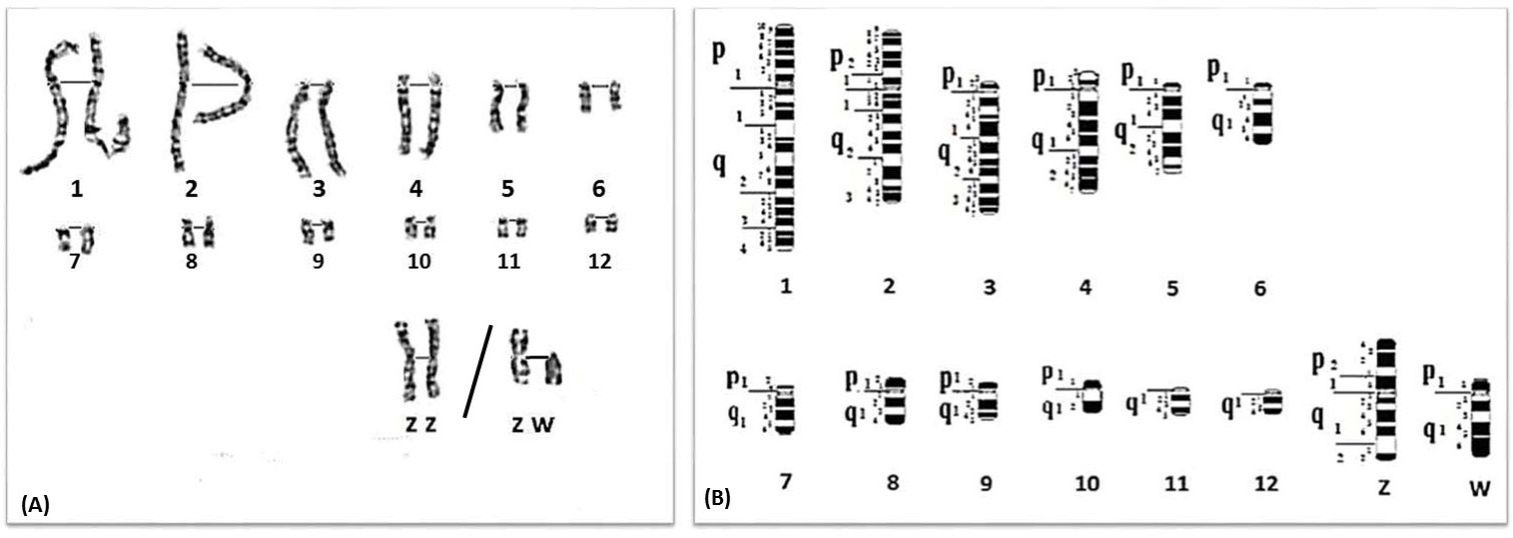
**A** GTG-banded karyotype for pairs 1 to 12 and sex chromosomes of the Japanese quail *Coturnixjaponica*. **B** Idiogram corresponding to **A**.

The GTG-banded karyotype and corresponding idiogram of the Japanese quail are illustrated in Figures 3 (A and B). Chromosome 1 is submetacentric and characterized by a centromere bordered by two narrow positive bands (11p and 11q). The short arm of submetacentric chromosome 8 has a region with 2 G bands, a negative narrow subcentromeric band (11) followed by a wide positive band (12). The q arm has only one region. 4 G-bands are observed, one subcentromeric negative band (11) followed by two prominent positive bands (12 and 14) separated by a large negative band (13). The W chromosome is subtelocentric and is ranked at the fifth position. The patterns of the GTG bands show that the p arm has a region with a narrow subcentromeric positive band. The q arm has one region and 6 bands, a subcentromeric negative band (11) followed by three positive bands (12, 14 and 16) separated by two negative bands, a large (13) and a narrow (15).

**Table 2.** Morphometry of the first twelve macrochromosomes and gonosomes of the Japanese quail *Coturnixjaponica* (n=16) p: short arm. q: long arm. p+q: relative length. CI: centromeric index=p/(p+q) × 100.

Chromosomes	p(µm)	q(µm)	q/p	p+q(µm)	CI%
**1**	1.3	2.8	2.15	4.1	31.70
**2**	1.25	1.66	1.32	2.91	42.95
**3**	0.14	2	14.28	2.14	8.18
**4**	0.32	1.7	5.31	2.02	15.84
**5**	0.15	1.11	7.4	1.26	11.90
**6**	0.08	0.76	9.5	0.84	9.52
**7**	0.1	0.66	6.6	0.76	13.16
**8**	0.24	0.47	1.95	0.71	33.80
**9**	0.07	0.56	8.56	0.63	11.11
**10**	0.09	0.49	5.44	0.58	15.51
**11**	0.08	0.46	5.75	0.54	14.81
**12**	0.05	0.48	9.6	0.53	9.43
**Z**	0.96	1.05	1.09	2.01	47.76
**W**	0.18	0.92	5.11	1.1	16.36

### GTG-banding patterns of chimeric hybrids and gynandromorphism

**Figure 4.**
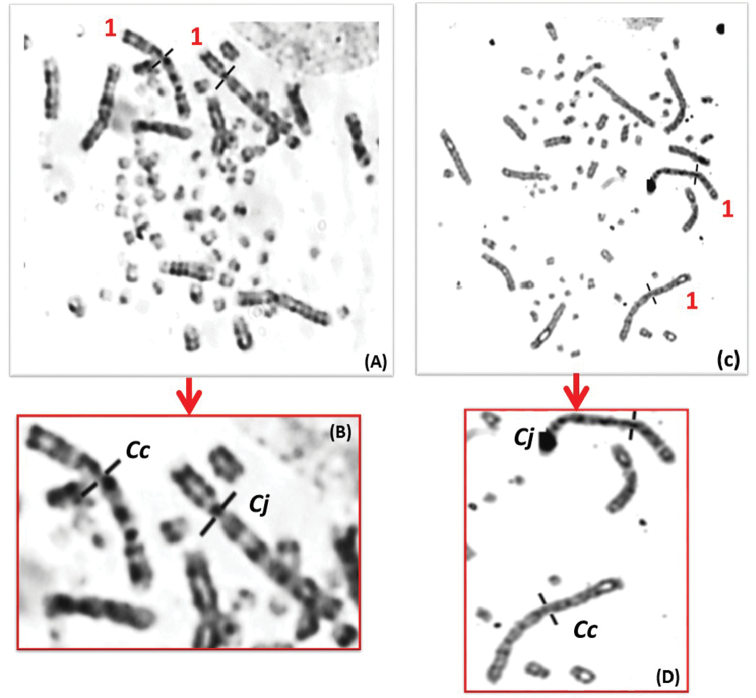
**A, C** Prometaphase spreads following the GTG-banded chromosomes of hybrid quail **B, D** Black traits indicate the centromere positions of the homologous chromosomes 1 which are morphologically different.

**Figure 5.**
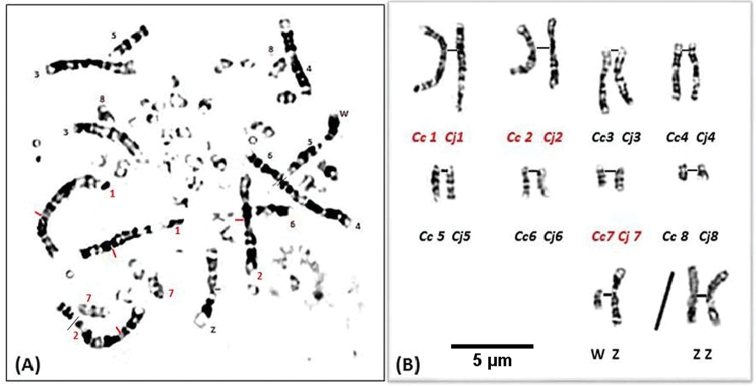
**A** Prometaphase spread following the GTG-banded chromosomes of hybrid quail **B** patterns of pairs 1 to 8 and sex chromosomes corresponding to **A** showing the differences on chromosomes 1, 2 and 7 of both species. Scale bar: 5µm.

Of the seven expected hybrid quails cultivated in this project, only two cell cultures have succeeded. These hybrids were analyzed in the 15^th^ generation, were all viable and derived from fertile parents. The homologous chromosomes of the same pair were designated “*Cc*” for Common quail and “*Cj*” for Japanese quail (Figure 4). The karyotype in GTG bands is shown in Figure 5 (A and B). Chromosome analysis of hybrid quails revealed morphological differences only on chromosomes 1, 2 and 7. The W chromosomes of both species are morphologically similar but a difference in size was observed (the W*Cj* is bigger than the W*Cc*) (Table 1 and 2).

**Figure 6.**
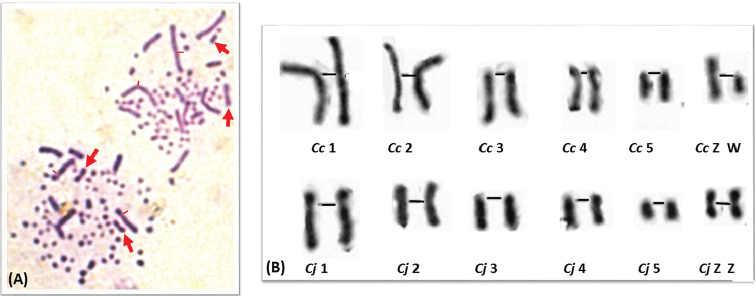
Chimera embryo showing **A** the cohabitation of the Common and Japanese quail cells **B** gynandromorphism corresponding karyotypes to **A** with ZZ/ZW chromosomes indicated by the arrows.

The two analyzed hybrid embryos showed a coexistence of three cell types that we have identified as chimeric hybrids (Figure 6A). In fact, a predominance of Japanese quail cells was observed (90%) whereas the cells of Common quails and hybrids were rarer (4% and 11% respectively). It is supposed to be a micro-chimerism.

Another anomaly has also been detected concerning sex chromosomes, which is a kind of chimerism called gynandromorphism. Thus, one of the two chimeric preparations corresponded to a gynandromorphic individual that corresponds to the presence of two distinct cell populations at a same time: male and female (Figure 6B). This embryo showed ZZ Japanese quail cells in addition to ZZ and ZW Common quail cells and hybrid cells with different sexual formulas. This embryo could be the result of a double fertilization of a female hybrid quail type (Z *Cj* / W *Cc*) by two males, Japanese and hybrid quail. The second embryo analyzed exhibited Common quail cells, in addition to Japanese and hybrid. They are all female cells, which could be the result of a cross between a female hybrid quail (Z *Cc* / W *Cj*) with two hybrid quail males.

### Comparative cytogenetic data from the Common quail, Japanese quail and domestic fowl

The chromosome comparison by GTG banding analysis of three species (Common quail, Japanese quail and Chicken *Gallusgallusdomesticus* “GGA”) confirms the presence of chromosomal rearrangements already described for Japanese quail and Chicken (Sasaki 1981, Stock and Bunch 1982, Shibusawa et al. 2001, 2004, Zlotina et al. 2012). Indeed, similarities between the three species have been observed on most macrochromosomes (3, 5, 6, 9, 10, 11 and 12). In our materials, the presence of chromosomal rearrangements on chromosomes 1, 2, 4, 7, 8 and W was noticeable. The Z chromosomes are morphologically similar in all three species (metacentric) and for which no inversion has been detected.

**Figure 7.**
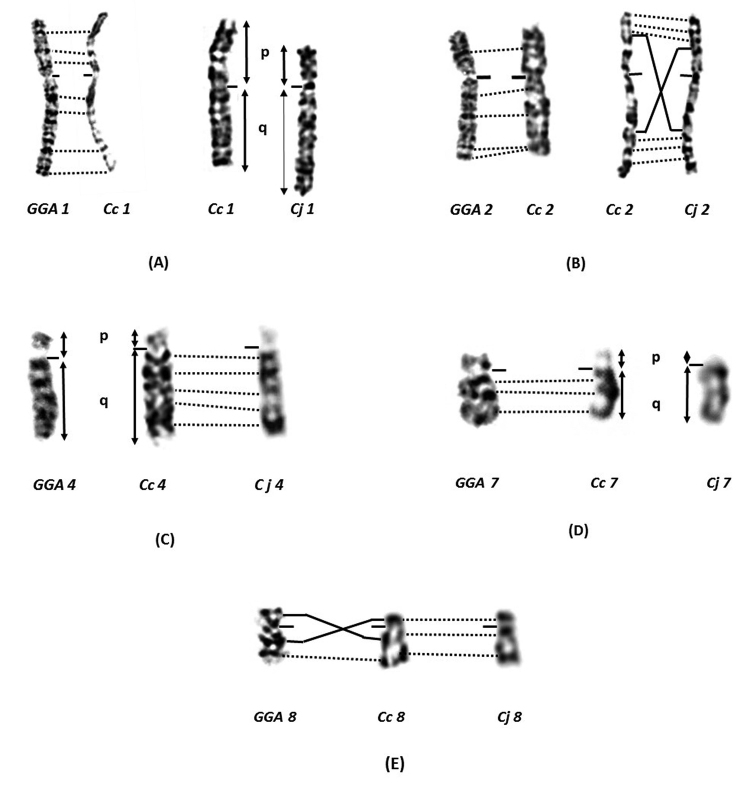
Comparison of chromosomes 1 **A** 2 **B** 4 **C** 7 **D** and 8 **E** of domestic chicken *GGA* (left), Common quail *Cc* (in the middle) and Japanese quail *Cj* (right) with the GTG bands.

Important homology was observed on chromosome 1 of the Common quail compared to its homologs in domestic chicken, while a perfect correspondence of the GTG band profiles is observed on chromosomes 1 of the two quail species, a difference in the ratio q/p was found (Figure 7A). Chromosome 2 of the Common quail and its Chicken homolog are very conserved. Some GTG band patterns of chromosomes 2 are completely reversed between the two quail species (Figure 7B). The ratio q/p of chromosomes 4 of the Common quail and Chicken is different (Figure 7C). A short p-arm visible and measurable on chromosome 7 of the Common quail looks more similar to its Chicken homolog than to the Japanese quail (Figure 7D, Table 2). The chromosome 8 of the Common and Japanese quails is morphologically similar. On the contrary, banding patterns differences in homologue chromosome 8 of chicken were observed (Figure 7E). The W chromosomes of the Common and Japanese quails exhibit strong homology (Figure 2 and 3), unlike the Chicken chromosome W (submetacentric). However, the size of the Common quail chromosome W is close to chromosome 7 whereas that of Japanese quail is close to chromosome 5 (Table 1 and 2).

## Discussion

Even though the cells of birds remain among the most difficult species to maintain in culture, the prometaphase cells are particularly suitable for bird analysis because the chromosomes are thin and elongate, making the structure of the smaller elements more distinct (Owen 1965, Ladjali et al. 1995, Ladjali-Mohammedi et al. 1999) .

The high sensibility observed in cells cultures of wild quail corroborate with the vulnerability of this species in breeding areas unlike the Japanese quail because of its easy practical prolificacy in captivity (Caballero de la Calle et Peña Montañés 1997, personal communication of the Tlemcen Hunting Centre). This is the case for the Barbary and Chukar partridges (Ouchia-Benissad and Ladjali-Mohammedi 2018).

The diploid number of 2N=78 estimated in Common quail and then in Japanese quail, emphasizes the exceptional conservation of karyotypes in the order of Galliformes (Ohno et al. 1964, Takagi and Sasaki 1974, Stock and Bunch 1982, Arruga et al. 1996, Shibusawa et al. 2004, Ishishita et al. 2014, Ouchia-Benissad and Ladjali-Mohammedi 2018). This is the case for the domestic fowl *Gallusgallusdomesticus*, too (Ladjali 1994).

The karyotypes of Common and Japanese quail show 8–10 pairs of macrochromosomes and 30–28 pairs of microchromosomes which are very difficult to distinguish. This is quite similar to that in most Galliformes (Stock and Bunch 1982, Shibusawa et al. 2004).

While the GTG band karyotype of the Japanese quail *Coturnixjaponica* was described up to the eighth chromosome pair only (Talluri and Vegnil 1965, Turpin et al. 1974, Ryttman and Tegelström 1981, Sasaki 1981, Stock and Bunch 1982, Shibusawa et al. 2001), in this study we have managed to describe up to the first 12 pairs and sex chromosomes. However, we have detected the presence of some ambiguities on the idiograms of chromosomes 1, 8 and W already proposed (Stock and Bunch 1982, Shibusawa et al. 2001). Indeed, the centromeric region of chromosome 1 is bounded by two positive G-bands that are characteristic of chromosome 1 of the Japanese quail (Figure 3 A, B). The result of chromosome 8 obtained in this work (Table 2) is supported by a previous studies (Talluri and Vegnil 1965, Stock and Bunch 1982), while other authors have described it as acrocentric (Schmid et al. 1989, Shibusawa et al. 2001). The description of the W chromosome corroborates with that of Hartung and Stahl (1974) and Schmid et al. (1989). Chromosomes W and 5 can be confused by size (Talluri and Vegnil 1965).

Comparative chromosomal analysis of both quails with domestic chicken allowed us to discover high conservation as well as differences in the karyotypes. The Common quail karyotype shares more similarities with chicken chromosomes than that of Japanese quail with Chicken. However, the Chicken karyotype is considered as the most similar to the putative ancestral bird karyotype (Griffin et al. 2007). The results obtained in this study suggest that, during speciation, Common quail would make an intermediate species between Chicken and Japanese quail.

**Figure 8.**
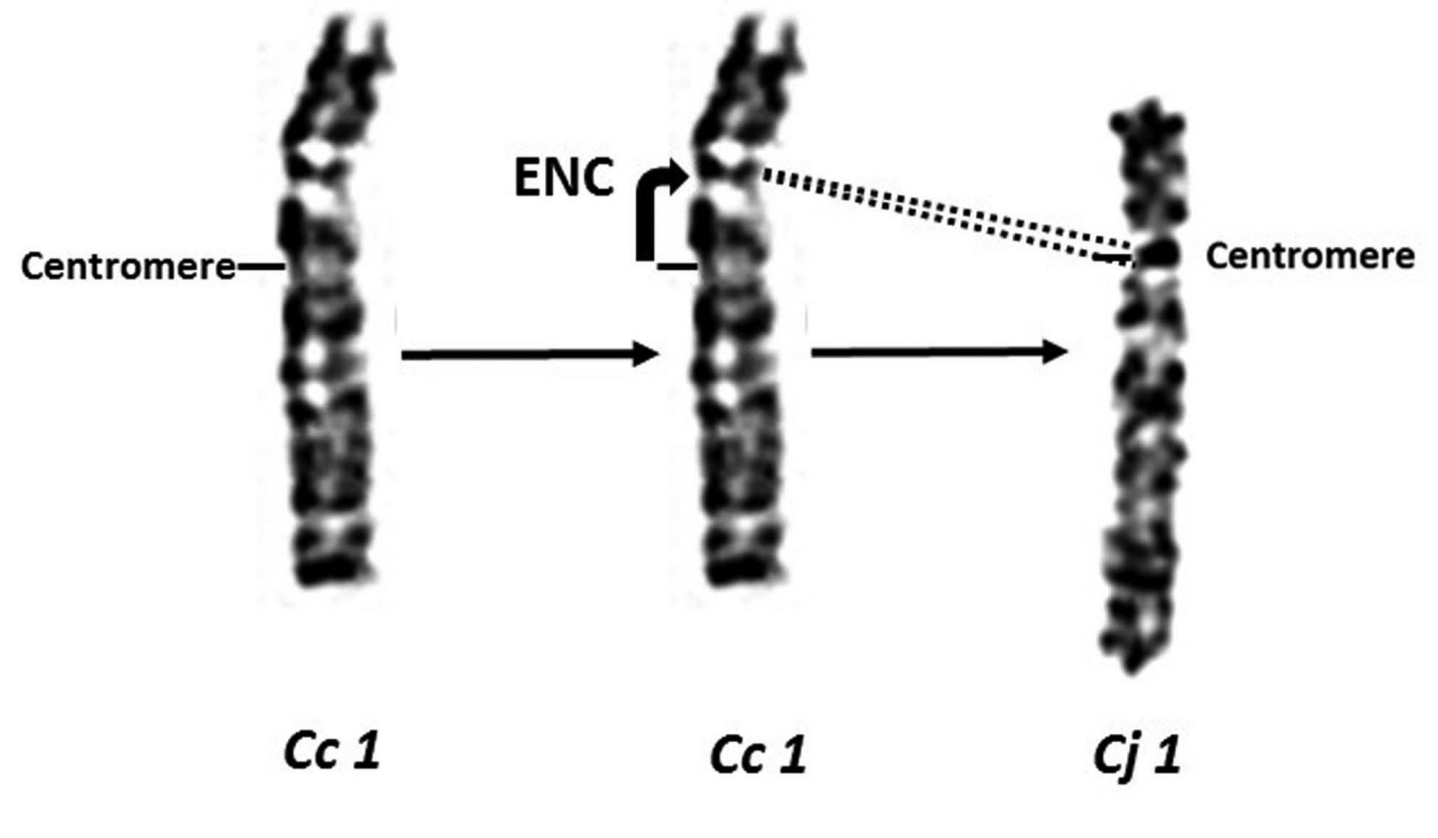
Evolutionary new centromere (ENC) formation on chromosome 1 of the Common and the Japanese quails.

In fact, the high conservation of GTG banding patterns of chromosomes 1 in these three Galliformes species is observed, whereas difference in the q/p arm ratio is detected on chromosomes 1 of the two quails. This result could be explained by a formation of an evolutionary new centromere (ENC) (Figure 8). The pericentric inversion hypothesis is therefore not verified in this work (Sasaki 1981, Stock and Bunch 1982, Kayang et al. 2006). This formation of a neocentromere would be more plausible and would correspond to the work of comparative mapping on Japanese quail meiotic chromosomes (Galkina et al. 2006, Zlotina et al. 2010, 2012).

**Figure 9.**
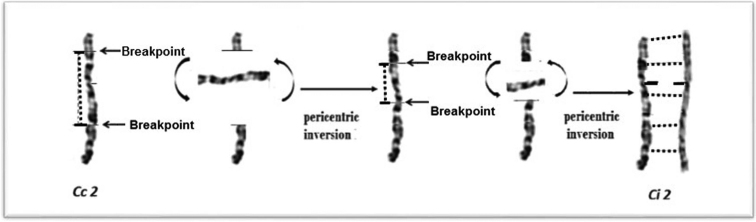
Double inversion that could have occurred during evolution on chromosome 2 between the Common and the Japanese quails.

Double pericentric inversion is demonstrated in some G-band motifs when chromosome 2 of Common quail and Japanese quail are compared as was reported in previous studies (Figure 9) (Shibusawa et al. 2001, Schmid et al. 2005, Kayang et al. 2006, Zlotina et al. 2012). The breakpoints on chromosome 2 of the Common quail would be located in the region between the band p 2.3 → q 3.1 and the band p 1.3 → q 1.4 (Figure 2A).

In the present work, we observed perfect conservation patterns in chromosome 4 of the three species. Furthermore, a morphological difference was noted between Chicken and the both quails. This result could suggest repositioning of the centromere during the speciation event (Figure 7C). This was already reported by Galkina et al. (2006) showing a perfect conservation of Chicken BAC clones order on Japanese quail chromosome 4. In fact, centromeres appear to be formed *de novo* during the evolution of Galliformes karyotypes (Kasai et al. 2003, Galkina et al. 2006, Skinner et al. 2009, Ouchia-Benissad and Ladjali-Mohammedi 2018).

However, the fourth avian chromosome pair is quite complex in the history of bird evolution (Chowdhary and Raudsepp, 2000). Multiple hypotheses were proposed to explain the differences in chromosome 4 of Japanese quail and domestic Chicken (Fillon et al. 2003, Schmid et al. 2005, Shibusawa et al. 2001, Galkina et al. 2006). Nevertheless, Chicken chromosome 4 is suggested to have arisen from a fusion of ancestral acrocentric chromosome 4 and ancestral microchromosome 10 (Schmid et al. 2000, Shibusawa et al. 2004, Griffin et al. 2007).

Comparative chromosome 7 mapping of Common quail highlighted a large conservation with domestic fowl (Figure 7D). However, deletion of the short arm p would have occurred in the common ancestor of Common and Japanese quail during evolution. We plan to locate molecular markers (chicken-specific BAC clones) that flank the centromere of chromosome 7 to confirm or reverse this type of rearrangement (Shibusawa et al. 2001, Fillon et al. 2003).

In both quails, the 8 chromosomes were highly similar but differences in the disposition of the GTG bands were observed comparing them to Chicken (Figure 7E). This would probably be the result of a pericentric inversion involving the region of the band p 1.1 and the band q 1.2 (Figure 2 B). These results confirm what has already been reported in Japanese quail (Shibusawa et al. 2001, Fillon et al. 2003, Sasazaki et al. 2006).

We observed high conservation between the Z chromosomes of the Chicken and the two quails. This result suggests no presence of pericentric inversion in the common ancestor of the three species, as previously described by Suzuki et al. (1999).

However, W chromosomes of both quails presented similarities. They have a small short arm, unlike the longer one in the Chicken. This morphological difference could be the result of formation of neocentromere (ENC) during the evolution. Moreover, the difference in size observed in the two species of quails could be explained by the fact that the ZW sex chromosomes would undergo unequal condensation/decondensation of the chromosomal arms (Solovei et al. 1993, Saifitdinova et al. 2003).

The observed differences between the Common and Japanese quail chromosomes dealt with chromosomes 1, 2 and 7. All of the rearrangements described probably occurred in the evolutionary process before the separation of the two quail species. The important chromosomal similarity between these two species could allow to obtain a fertile and highly prolific progeny (Asmundson and Lorenz 1957, Hidas 1993). Also, sterility was shown to be related to the presence of large blocks of heterochromatin in the hybrids chromosomes (Wójcik and Smalec 2017). It was not observed on chromosomes of the hybrid embryos that we analyzed.

The presence of different cell types (*Cc* and *Cj*) within the same hybrid individual may be due to double fertilization of the ovule and its polar globule from sperms of different origins. Indeed, surviving spermatozoa from anterior mating can be preserved in the female genital tract at the infundibulum and could then be released into the oviduct lumen (Grigg 1957). The results we obtained can also support the theory of parthenogenesis which is an asexual reproduction without fertilization, exclusive to females (Servella 1974).

Chimerism is an extremely rare abnormality in animals. The proportions of the three cell types obtained represented a micro-chimerism which is defined by the number of cells affected. It is when a genetically foreign population represents less than 5% of the nucleated cells of an individual or organ (Nelson 2010). Similar observations were made over chicken-pheasant hybrids (Basrur and Yamashiro 1972). It has already been observed that females of Japanese quail breed with Japanese wild or hybrid quail males (Guyomarc’h et al. 1998, Collins and Goldsmith 1998). While Common quail females mate mainly with males of the same species (Guyomarc’h 2003, Domjan et al. 2003, Dérégnaucourt et al. 2005, Sanchez-Donoso et al. 2012, Puigcerver et al. 2014). These data corroborate with those obtained in this study.

Gynandromorphism is an anomaly that is not very well answered in birds (Gilgenkrantz 1987). Only one gynandromorph individual was analyzed. Some cases were observed in a red cardinal and chickens (Peer et al. 2014). Gynandromorphism would be the result of a genetic mutation occurring during the early division of the oocyte after fertilization (Hollander 1975). Fusion of two eggs that should have given a male and a female would give birth to individuals with both cells of different sexes (Zhao-Xian et al. 2010, Clinton et al. 2012).

## Conclusion

The analysis of hybrid animals bred in western Algeria showed us that introgressive hybridization affected the genetic heritage of the Common quail *Coturnixcoturnix* and would be a threat to its preservation. Although the wild quail and Japanese quail are phylogenetically very close, the chromosome banding method allowed us to propose the karyotype of the Common quail and to distinguish these two taxa. The comparative cytogenetic study allowed us to detect ancestral intrachromosomic rearrangements that could have accompanied the speciation and evolution of the karyotypes of the three species of Galliformes. Common quail would be an intermediate species between the Chicken and Japanese quail, which would be more recent in appearance. As a result, cytogenetics is a very important element in taxonomy and phylogeny studies.

In addition, for better knowledge of the Common quail genome, Fluorescence *In Situ* Hybridization (FISH) will be performed for individual microchromosome identification (Lithgow et al. 2014, McPherson et al. 2014). Though specific FISH probes of GGA11-28 chicken lampbrush microchromosomes can be used for the 10 smallest chicken microchromosomes, GGA29-38, no individual molecular tags have been established to date (Galkina et al. 2017, Kretschmer et al. 2018). Also, the characterization of the nuclear genetic markers (microsatellites) allowed to distinguish both taxa of the quail and their hybrids, and to estimate the genetic introgression (Boecklen and Howard 1997, Puigcerver et al. 2000, Rodríguez-Teijeiro et al. 2003, Barilani et al. 2005, Vähä and Primmer 2006, Chazara et al. 2006, 2010). Finally, microdissection of chromosomes or large-scale sequencing could enable us to refine the knowledge of specific microchromosomal regions (Fillon 1998, Masabanda et al. 2004).
